# Efficacy of praziquantel in the treatment of *Schistosoma haematobium* infection among school-age children in rural communities of Abeokuta, Nigeria

**DOI:** 10.1186/2049-9957-3-30

**Published:** 2014-09-01

**Authors:** Olusola Ojurongbe, Olawunwi Risqat Sina-Agbaje, Abass Busari, Patricia Nkem Okorie, Taiwo Adetola Ojurongbe, Akeem Abiodun Akindele

**Affiliations:** 1 Department of Medical Microbiology and Parasitology, Ladoke Akintola University of Technology, Osogbo, Nigeria; 2 Department of Medical Microbiology and Parasitology, General Hospital, Ijaye, Abeokuta, Nigeria; 3 Department of Medical Laboratory Services, Olikoye Ransome-Kuti Hospital, Abeokuta, Nigeria; 4 Institute for Advanced Medical Research and Training, College of Medicine, University of Ibadan, Ibadan, Nigeria; 5 Department of Mathematical and Physical Sciences, Osun State University, Osogbo, Nigeria

**Keywords:** Urinary schistosomiasis, Praziquantel, Drug efficacy, School children, Nigeria

## Abstract

**Background:**

Chemotherapy with praziquantel (PZQ) has been the cornerstone of schistosomiasis control over the last two decades. Being the only available drug for the treatment of over 200 million people worldwide, continuous monitoring of PZQ efficacy under the pressure of widespread use is therefore advocated.

**Methods:**

The efficacy of taking two doses of oral PZQ for the treatment of *Schistosoma haematobium* was examined among school children in Nigeria. Urine specimens were collected from 350 school children and examined using the filtration technique. Blood was collected for packed cell volume (PCV) estimation, and the weight and height of each child were estimated. *S. haematobium* egg positive pupils were treated with two oral doses of PZQ at 40 mg/kg with a four-week interval in between. Drug efficacy was determined based on the egg reduction rate (ERR).

**Results:**

Among 350 school children, 245 (70.0%) – of which 132 were males and 113 were females, with an age range of 4 to 15 years – were diagnosed with *S. haematobium.* All the 245 infected children received a single oral dose of 40 mg/kg PZQ twice with a four-week interval in between and were followed up for 12 weeks. At four, eight and twelve weeks post treatment, the ERR was 57.1%, 77.6% and 100%, respectively. The ERR was significantly higher among the children with a light infection compared to those with a heavy infection. One hundred and twenty-one children were egg negative at four weeks post treatment, among which 1 (6.3) and 120 (52.4%) had heavy and light infections, respectively. Following the second round of treatment, the cure rate at eight weeks and twelve weeks was 85.3% and 100%, respectively.

**Conclusion:**

This study demonstrated the efficacy of taking two doses of oral PZQ for the treatment of urinary schistosomiasis among school children in Nigeria.

## Multilingual abstracts

Please see Additional file 1 for translation of the abstract into the six official working languages of the United Nations.

## Background

Schistosomiasis is responsible for significant health problems and is a socioeconomic burden in most of Sub-Saharan Africa and some other tropical countries [1,2]. It is endemic in 74 countries, with the bulk of the cases globally (90%) residing in Sub-Saharan Africa [3,4]. The significant health problems associated with schistosomiasis include impaired cognitive potential among primary school-age children, hepatosplenomegaly, anaemia, bladder cancer and stunted growth [5]. Also genital schistosomiasis, which manifests at reproductive age if schistosomiasis is not treated, is attributed as a risk factor for HIV transmission [6].

Currently the antischistosomal drug of choice for the treatment of schistosomiasis is praziquantel (PZQ). It is the mainstay of the current strategy against schistosomiasis morbidity control and is highly effective against the five schistosome species that infect humans [7]. Praziquantel is a pyrazinoquinoline derivative and its safety and efficacy have ensured its widespread usage. It is recommended that school-aged children and high-risk groups of adults in communities with a prevalence of 10% to 50% use it once every two years. In communities where the prevalence is above 50%, both children and adults are required to be treated once a year [8]. Praziquantel has been used widely successfully in many national control programmes. However, there is evidence of clinical relevant resistance developing [9].

The extensive use of PZQ and the problem of reduced therapeutic efficacy is on the increase globally, and this has led to the growing concern regarding the use of a single drug for the treatment of a disease affecting more than 200 million people [10]. Already countries such as Egypt [11], Zimbabwe [5] and Cameroon [12] have reported low cure rates of PZQ. There is therefore a need for constant and continuous monitoring of PZQ under the pressure of widespread use. A critical aspect in the assessment of PZQ efficacy is the drug’s activities during the different parasite development stages. Experimental laboratory studies have shown that activities of PZQ are stage dependent with the drug acting primarily against the adult worm stages, whereas immature schistosomes (two to four weeks old) are less susceptible [13]. All these observations point towards the need for the development of new potent drugs or combinations of drugs that will be effective against all stages of the parasite. While this is still being awaited, there is a need for constant monitoring of PZQ efficacy in endemic areas.

Although the prevalence and incidence of *S. haematobium* has been largely reported in Nigeria [14-16], the efficacy of PZQ among school children has not been well documented in some highly endemic areas as the drugs are distributed on a large scale without monitoring or follow-up. Therefore, this study was conducted to evaluate the efficacy of taking two doses of oral PZQ for treatment, given as two single doses of 40 mg/kg with a four-week interval in between. Repeated doses of PZQ given two to eight weeks after the initial dose in endemic areas of Africa have been shown to have incremental benefits when compared to single doses [17,18].

## Methods

### Study site and subjects

This study was carried out among primary and secondary school children in Akala and Imala Odo (Oyan) in the Abeokuta North Local Government area of Ogun State between July 2011 and March 2012. The communities were located close to the bank of the Oyan Reservoir which has been shown to be endemic [19]. The communities had no health centre at the time of the study. Although there is a schistosomiasis co-ordinating unit at the local government council, its activity is hampered by the irregular supply of PZQ. The study enrolled 350 primary and secondary school children, aged four to fifteen years. The age of each child was determined based on school records, and the weight and height were measured using a ruler and a weighing scale, respectively.

### Screening of urine

Universal bottles labelled with the corresponding identification number were given to each participating child. Each participants was asked to produce the urine specimen between 10:00 and 14:00, and submit it on the same day. The samples were transported to the laboratory for microscopic examination of *S. haematobium* eggs using the urine filtration method. Briefly, 10 ml of well-mixed urine was aspirated and slowly forced through a filter membrane. The filter was removed and placed on a slide, covered with a cover slip and examined under a light microscope [20]. The number of eggs on the entire filter was counted and recorded as the number of eggs per 10 ml urine (EP10ml). From the total slides, 10% were randomly selected and re-examined by an independent microscopist for quality control. A Combur-Test (Roche Diagnostics GmbH, Mannheim, Germany) reagent strip was used to detect the presence of blood in the urine. For those participants whose slides came out negative, urine samples were collected on two successive days to confirm that they were truly negative.

### Determination of packed cell volume

For packed cell volume (PCV), microhematocrit tubes filled with blood were centrifuged in a microhematocrit rotor for five minutes at 10,000 g. Packed cell volumes values ≤ 31% were considered as anaemia, which was further classified as mild (21–30%), moderate (15–20%) or severe (≤15%).

### Treatment and follow-up

All children who provided urine specimens in the pre-treatment survey were included in the analysis of infection patterns at baseline, but only the children positive for *S. haematobium* eggs were treated with two single oral doses (40 mg/kg) of PZQ, given with a four-week interval in between. The drug was administered with a glass of water following the confirmation that the child ate at home or ate food that was provided by the investigating team. Urine samples were taken on the fourth, eighth and twelfth week to monitor the cure and egg reduction rates.

### Ethical consent

Approval for the study was obtained from the Ethical Committee of the Ogun State Ministry of Health in Abeokuta. Before the onset of the study, a meeting was held with the community heads and the parents of the school children where formal, oral informed consent was obtained from the parents and the children directly before the sample collection.

### Data analysis

Data was entered into a Microsoft Excel spreadsheet and exported to SPSS (version 16) (SPSS Inc., Chicago, IL, USA) for analysis. The proportion of children infected with *S. haematobium* was expressed as prevalence. The chi-square test and one-way ANOVA were used to test for differences in the prevalence of infections and geometric mean egg counts, respectively. P-values < 0.05 were considered statistically significant. The intensity reduction rate was calculated as [1 - (GM egg counts per 10 mL of urine after treatment/GM egg counts per 10 mL before treatment)] × 100 [21]. The cure rate was calculated as [the number of children excreting no *S. haematobium* eggs/the number of children with confirmed infections before treatment] x 100.

## Results

Of the 350 children (age range four to fifteen years; 186 male, 164 female) enrolled into the study, 245 (70.0%) – 132 males and 113 females – were positive for *S. haematobium*, determined using the urine filtration technique. The age group of four to nine years had the highest prevalence (70.4%), but the difference was not statistically significant. There was no significant difference in the intensity of infection according to sex or age in the study. The proportion of males (5.9%) that had a heavy infection was greater than females (3.1%), but the difference was not statistically significant. The age group of four to nine years had the highest proportion (5.4%) of those with a heavy infection but this difference was also not statistically significant (see Table 1).

**Table 1:** Prevalence and mean egg count/10 ml urine, by sex and age, among 350 school children in the Abeokuta North Local Government area

***Sex***	***No Examined N = 350***	***No. Positive (%)***	***No. Heavy (%) (≥50 egg/10 ml)***	***No. Light (%) (<50 egg/10 ml)***	***Geometric mean egg count/10 ml***
Male	186	132 (70.9)	11 (5.9)	121 (65.1)	10.98
Female	164	113 (68.9)	5 (3.1)	108 (65.9)	7.37
p-value		0.73		0.30	0.91
***Age (years)***					
4–9	203	143 (70.4)	11 (5.4)	132 (65.0)	7.93
10–15	147	102 (69.4)	05 (3.4)	97 (66.0)	6.73
p-value		0.90		0.67	0.72

**Figure 1:**
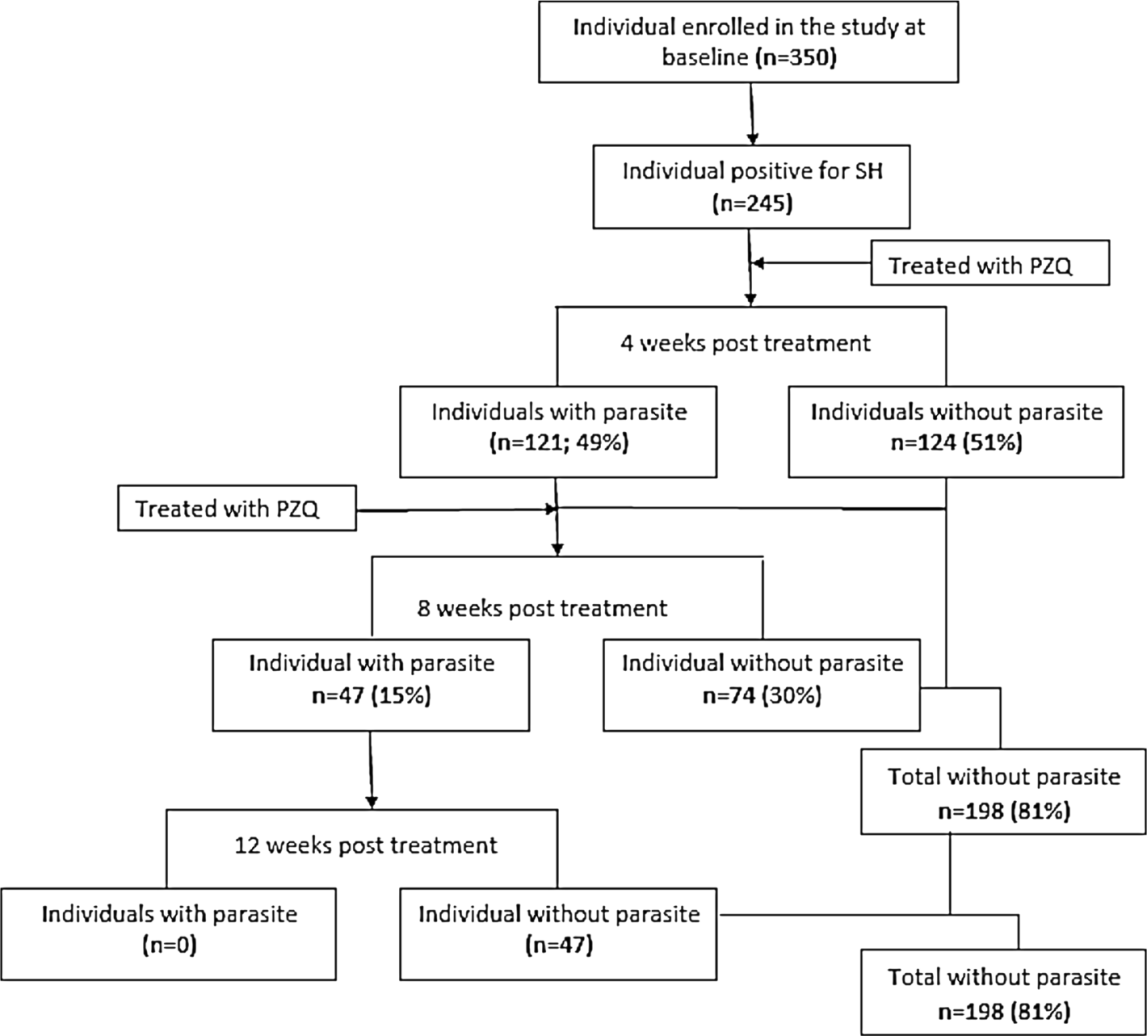
**Study compliance for the efficacy of praziquantel to treat *S. haematobium *** **among school children in Abeokuta, Nigeria.**

Figure 1 shows the operational results and PZQ treatment outcomes over a 12-week period. Two hundred and forty-five children were treated twice (with a four-week interval in between) over a 12-week period with follow-up. All 245 infected children received a single oral dose of 40 mg/kg of PZQ and another single dose of 40 mg/kg four weeks later under the supervision of their teachers and members of the study team. The overall ERR at four weeks, eight weeks and 12 weeks post treatment was 57.1%, 77.6% and 100%, respectively. The efficacy of PZQ in this study at 12 weeks post treatment is therefore considered satisfactory [21]. The cure rate at four weeks, eight weeks and twelve weeks was 49.4%, 85.5% and 100%, respectively.

Before treatment, 6.5% (geometric mean egg 116.4 egg/gm) of the children were heavily infected (>50 eggs/10 ml), while 93% (geometric mean egg 8.2egg/gm) were lightly infected. At four weeks post treatment (before the second dose of PZQ), only 1 (6.3%) child was parasite free among those who were heavily infected. There was a significant reduction between day 0, and four, eight and twelve weeks in terms of the mean egg count (p = 0.0001). At eight weeks and twelve weeks (after the second dose of PZQ), 7 (43.8%; ERR 96.4%) and 16 (100%; ERR 100%) were parasite free, respectively, among those who were heavily infected. For those who were lightly infected, the ERR at four weeks, eight weeks and twelve weeks was 58.9%, 73.1% and 100%, respectively. The drug showed an overall cure rate of 49.4%, 85.3% and 100% at four weeks, eight weeks and twelve weeks, respectively (see Table 2).

**Table 2:** **Cure rates, geometric mean egg counts and intensity reduction rates over 12 weeks in 245 school children infected with *Schistosoma haematobium *** **after one treatment with praziquantel in Ogun State, Nigeria**

	***Before treatment***		***4 weeks after treatment***				***8 weeks after treatment***				***12 weeks after treatment***			
**Egg Intensity (egg/10 ml)**	***No. of subjects (%)***	***GM egg/ 10 ml***	***No. cured***	***Cure rate (%)***	***GM eggs/ 10 ml***	***Egg reduction rate (%)***	***No. cured***	***Cure rate (%)***	***GM eggs/ 10 ml***	***Egg reduction rate (%)***	***No. cured***	***Cure rate (%)***	***GM eggs/ 10 ml***	***Egg reduction rate (%)***
< 50	229 (93.5)	8.2	120	52.4	3.37	58.9	199	87.0	2.2	73.1	229	100.0	0.0	100.0
≥ 50	16 (6.5)	116.4	1.00	6.3	13.4	88.5	7.0	43.8	4.2	96.4	16	100.00	0.0	100.0
**Total**	245 (100)	9.8	121	49.4	4.2	57.1	206	85.3	2.2	77.6	244.0	100.00	0.0	100.0

Generally none of the pupils examined in the study population, either infected or non-infected with schistosomiasis, were anaemic. A significant difference was observed when the level of infection was compared with the mean PCV in the study population (p = 0.017). The mean weight and height of the pupils were also compared with respect to the infection level but the difference was not significant. A significant difference was observed when the presence of urine in the blood was compared with the different levels of infection (see Table 3). At the baseline cross-sectional survey, 228 of the 245 *S. haematobium* infected children had visible blood in their urine with a prevalence of 93.0%. At four weeks post treatment, only 2 (1.2%) children had blood detected, while none of the children had blood in their urine by the eighth and twelfth week.

**Table 3:** **Comparison between PCV, weight, height and blood in urine in relation to *S. haematobium *** **intensity of infections among school children**

**Characteristics**	***No infection (0 EP10ml) N = 105***	***Light infection (<50 EP10ml) N = 229***	***Heavy infection (≥50 EP10ml) N = 16***	***P-value***
*PCV (geo mean) ± SD*	34.21 ± 2.7	33.86 ± 2.6	35.7 ± 2.5	0.017
*Weight (mean) ± SD*	23.57 ± 7.5	23.42 ± 7.3	25.50 ± 5.2	0.97
*Height (geo mean) ± SD*	116.73 ± 18.7	116.24 ± 20.5	114.94 ± 22.7	0.94
*Blood in urine (%)*	17 (16.2)	212 (92.6)	16 (100)	< 0.000

## Discussion

Praziquantel remains the drug of choice for the treatment of schistosomiasis in spite of cases of low cure rates that have been reported in some areas [22,23]. As there have been reports of therapeutic failures [24,25], constant surveillance of PZQ efficacy is imperative while we wait for the discovery and development of more potent drugs. In this study, the performance of PZQ was considered satisfactory since the ERRs during the eighth and twelfth weeks were 77.6% and 100%, respectively [21]. Our results also demonstrated that there is a significant difference between the cure rates and infection intensities which is in agreement with previous studies where cure rates were shown to be consistently higher in individuals with light infections before treatment than in those with moderate or heavy infections [17,26]. In some studies, factors such as high pre-treatment egg intensities, poor drug absorption and a high rate of PZQ catabolism, rather than parasite resistance, have been attributed to the reduced PZQ cure rate in endemic areas [5]. The criteria of cure based on the absence of eggs after treatment with PZQ have shown a variation of 60% to 96% depending on the intensity of the infection. At four weeks post treatment before the administration of the second single dose of 40 mg/kg, only 49.3% of the study population treated were parasite free. At eight weeks post treatment, after the second dose of PZQ, 15% of the treated population were still egg positive. Most of the previous studies have reported a higher efficacy of PZQ when administered as two or three treatments spaced at certain time intervals [23,27]. It is assumed that patients in high transmission areas would harbour a high number of immature schistosomes that are less susceptible to PZQ [28,29]. These immature schistosomes have a high chance of surviving a single PZQ treatment. Another explanation is the suggestion that PZQ kill worms slowly or that dead worms still have the ability to release eggs [12]. The dead eggs may be present for months in the urine of treated patients infected with *S. haematobium* and will falsely reduce the egg reduction and cure rate results. Although we did not determine the viability of the excreted eggs, some studies have reported that dead eggs may be present for several months in the urine of *S. haematobium* infected individuals [12].

The high prevalence of *S. haematobium* reported in this study reflects the current transmission situation and ongoing control challenges in this area. Many epidemiological studies emanating from this region over the years have consistently revealed a high prevalence of urinary schistosomiasis [14,30,15]. Some of these reports show that the community members are aware that their constant continuous contact with endemic water from the river is responsible for their high infection rates, but because there is no alternative water source they have no choice but to continue to depend on the contaminated water for their daily domestic activities [31]. An alternative source of water and a well-laid-out control strategy by policy makers coupled with regular PZQ treatment are therefore needed in other to put an end to the menace of urinary schistosomiasis in this region.

No significant difference was observed in the prevalence between males (70.9%) and females (68.9%), as well as between the different ages in this study. This pattern of infection may be an indication of the equal exposure of both genders and the ages examined in the study due to significant water contact activities. The observed prevalence of microhematuria (70%) in the overall study population was similar to what was previously reported in this study area [19]. No significant difference was observed regarding microhematuria between ages and sexes, and this may be explained by the fact that microhematuria is a characteristic symptom of urinary schistosomiasis in endemic communities and its prevalence has been shown to correlate positively with urinary schistosomiasis infection. This prevalence decreased rapidly during the fourth and eighth week following PZQ treatment. This finding is in agreement with previous studies that suggest that urine reagent strips could potentially estimate the intensity of *S. haematobium* infection in endemic areas [31,20]. The current WHO guidelines for preventive chemotherapy recognize hematuria prevalence, in addition to egg count-based criteria, as an effective means to identify communities with high, moderate or low risk for schistosomiasis [32].

An unusual result observed in our study was that none of the students infected with either a light or heavy *S. haematobium* infection were anaemic. We observed that the mean PCV of pupils with heavy infection intensity was significantly higher than pupils with a light infection or those who were not infected. Many previous studies have demonstrated a high prevalence of anaemia among school children living in *S. haematobium* endemic areas [33,16]. While anaemia is well associated with schistosomiasis based on the pathogenesis of blood loss that results from *S. haematobium* infection, the blood loss could also be compensated with foods such as fish and green leafy vegetables which are abundantly available and consumed in these local communities. The unexpected observation in this study could raise important questions that need further research. In a similar manner, the *S. haematobium* infection status has no influence on the weight and height of the students in this endemic area.

## Conclusion

This study demonstrated the efficacy of PZQ against *S. haematobium* in Nigeria. In view of the fact that the use of the drug will only increase as it is the only available widely-used drug, constant monitoring of drug resistance becomes imperative. It would also be beneficial to investigate the effects of repeated therapeutic doses of PZQ at different time intervals since repeated doses have been reported to produce a higher efficacy in comparison to single treatment in endemic areas. There is need to explore other alternative treatment regimens such the PZQ and artemisinin (ACT) derivatives combination as ACT derivatives have been shown to be active against immature schistosome worms [34]. There is need for a more co-ordinated effort by the government through the provision of alternative water sources that are cercariae free, through continuous and constant mass drug administration and through integrated vector control in order to bring an end to the ravaging scourge of this disease in this area.

## Competing interests

The authors declare that there are no competing interests.

## Authors’ contributions

OO designed, supervised and wrote the manuscript. S-AOR, BA and AAA participated in the collection of the sample, analysis of the sample and in drug administration and follow-up. OPN participated in manuscript preparation. OTA participated in the statistical analysis of the data. All authors read and approved the final manuscript.

## Supplementary Material

Additional file 1 Translation of the abstract into the six official working languages of the United Nations. Click here for file
