# Supplementary material for: Efficacy of praziquantel in the treatment of Schistosoma haematobium infection among school-age children in rural communities of Abeokuta, Nigeria
Source: Infect Dis Poverty. 2014 Sep 1;3:30. doi: 10.1186/2049-9957-3-30 (PMC4161270; doi:10.1186/2049-9957-3-30)
Supplement: Additional file 1 — Translation of the abstract into the six official working languages of the United Nations. [file 2049-9957-3-30-S1.pdf]

Translation of the abstract into the six official working languages of the United Nations

فعالية البرازيكوانتيل (**Praziquantel**) في علاج عدوى البلهارسيا الدموية بين الأطفال في سن التعليم في المجتمعات الريفية في أبيكوتا بنيجيريا.

أوجرونجبي أولسولا، وسينا أجباج، أولاوينوي رزقات، وبوساري عباس، وأكوري باتريشيا إن، وأوجرونجبي تاوه أديتولا، وأكينديايه أكيم أبيودن

ملخص

خلفية: العلاج الكيميائي باستخدام البرازيكوانتل (**Praziquantel**) هو العلاج الأساسي لمكافحة البلهارسيا على مدار العقدين السابقين؛ ونظرًا لأنه العقار الوحيد المتاح لعلاج أكثر من 200 مليون شخص على مستوى العالم، تستمر مراقبة مدى فعالية هذا العقار مع ضغوط انتشار استخدامه.

الطرق: اختُبِرت فعالية تناول جرعتان عن طريق الفم من البرازيكوانتيل (**Praziquantel**) لعلاج البلهارسيا الدموية بين أطفال المدارس في نيجيريا، حيث جُمِعت عينات بول من 350 طفل بالمدرسة وفُحِصت باستخدام تقنية التشريح وجمعت عينات دم لتحديد حجم الخلايا المكدسة. كان قد تم قياس وزن وطول كل طفل. بالنسبة للأطفال الذين كانت نتائج فحصهم إيجابية لوجود البيض، قُدِم لهم علاج عبارة عن جرعتان عن طريق الفم من البرازيكوانتيل (**Praziquantel**) بمقدار 40 مليجرام/كيلوجرام، يفصل بين كل جرعة وجرعة أربعة أسابيع، وحُدِدَت فعالية الدواء بناءً على معدل انخفاض البيض.

النتائج: من بين 350 طفل في المدرسة شُخِّص 245 -أي 70.0%- منهم 132 ولدًا ومنهم 113 فتاة، بمعدل عمر يتراوح بين أربعة إلى خمسة عشر عاما بعدوى البلهارسيا الدموية. تلقى جميع المصابين البالغ عددهم 245 جرعة عن طريق الفم، واحدة تحتوي على 40 مليجرام/كيلوجرام من البرزيكوانتيل (**Praziquantel**) مرتان يفصل بينهما أربعة اسابيع، وتمت متابعتهم لمدة 12 أسبوع. وبعد العلاج في الأسبوع الرابع والثامن والثاني عشر، كان معدل انخفاض البيض بنسبة 57.1% ونسبة 77.6% ونسبة 100% على التوالي، كما كان معدل انخفاض البيض أعلى بشكل ملحوظ بين الأطفال المصابين إصابة طفيفة مقارنة بأصحاب الإصابة الشديدة، حيث وصل عدد الأطفال الذين بينوا نتائج سلبية لوجود البيض إلى مئة وواحد وعشرين طفلًا بعد أربعة أسابيع من العلاج من بينهم واحد (6.3) ومئة وعشرون (52.4%) عانوا من إصابات شديدة وطفيفة على التوالي. وبعد الدورة الثانية من العلاج، وصل معدل الشفاء أثناء الثمانية أسابيع والإثنى عشر أسبوعًا إلى نسبة 85.3% ونسبة 100% على التوالي.

الخاتمة: أوضحت هذه الدراسة فعالية تناول جرعتان عن طريق الفم من البرزيكوانتيل (**Praziquantel**) لعلاج البلهارسيا البولية بين أطفال المدارس في نيجيريا.

Translated from English version into Arabic by Ahmad Hegazy, through

TRANSLATORS
WITHOUT BORDERS

吡喹酮治疗尼日利亚阿贝奥库塔乡村社区学龄儿童埃及血吸虫病的疗效

Ojurongbe Olusola, Sina-Agbaje Olawunwi Risqat, Busari Abass, Okorie Patricia N, Ojurongbe Taiwo Adetola, Akindele Akeem Abiodun

摘要

背景：吡喹酮化疗是近二十年来血吸虫防控的基石。作为治疗超过2亿人群的唯一抗血吸虫病药品，因此在该药被广泛使用的压力下，应倡导持续监测吡喹酮的疗效。

方法：在尼日利亚的在校儿童中，检测口服两次剂量吡喹酮治疗埃及血吸虫病的效果。采集350位在校儿童的尿样，使用过滤技术对尿液进行检测。采集血样用于红细胞压积检测，记录每位儿童的身高和体重。埃及血吸虫虫卵检测阳性的学生接受两次间隔为4周的吡喹酮治疗，每次剂量为40 mg/kg。以虫卵减少率（ERR）评价药物疗效。

结果：在 350 名在校儿童中，245 名（70.0%）被诊断为埃及血吸虫病患儿，其年龄范围为 4 到 15 岁。其中 132 名男童，113 名女童。所有患儿接受两次间隔为 4 周的吡喹酮治疗，每次剂量为 40 mg/kg，并继观 12 周。在治疗后 4 周、8 周、12 周，ERR 分别为 57.1%、77.6%和 100%。轻度感染患儿的 ERR 明显高于重度感染患儿。245 名患儿中，121 名患儿治疗 4 周后虫卵检测结果为阴性。在治疗前，该 121 名患儿中 1 名（6.3%）为重度感染，120 名（52.4%）为轻度感染。经第二轮治疗后，服药后 8 周和 12 周的治愈率分别为 85.3%和 100%。

结论：该研究阐述了两次口服吡喹酮治疗尼日利亚在校埃及血吸虫病患儿的疗效。

Translated from English version into Chinese by Zheng Qi, through

CHINESE CENTER FOR DISEASE CONTROL AND PREVENTION
NATIONAL INSTITUTE FOR PARASITIC DISEASES

Efficacité du praziquantel pour le traitement des infestations par *Schistosoma haematobium* chez les enfants d'âge scolaire dans les communautés rurales d'Abeokuta au Nigeria

Ojurongbe Olusola, Sina-Agbaje Olawunwi Risqat, Busari Abass, Okorie Patricia N, Ojurongbe Taiwo Adetola, Akindele Akeem Abiodun

Résumé

Contexte : Depuis deux décennies, la lutte contre la schistosomiase repose sur la chimiothérapie au praziquantel (PZQ). S'agissant du seul médicament disponible pour traiter plus de 200 millions de personnes dans le monde, il est nécessaire de contrôler en permanence son efficacité dans les conditions difficiles d'une utilisation étendue.

Méthode : L'efficacité de deux doses de PZQ par voie orale pour le traitement de *Schistosoma haematobium* a été étudiée chez des enfants d'âge scolaire au Nigeria. Des échantillons d'urine ont été recueillis auprès de 350 enfants scolarisés et examinés par filtration. Des échantillons de sang ont également été prélevés pour estimer l'hématocrite ; le poids et la taille de chaque enfant ont été estimés. Les écoliers positifs à la recherche d'œufs de *S. haematobium* ont été traités avec deux doses par voie orale de PZQ à 40 mg/kg espacées de quatre semaines. L'efficacité du médicament a été déterminée sur la base du taux de réduction du nombre d'œufs (TRO).

Résultats : Parmi les 350 écoliers, 245 (70,0 %, dont 132 garçons et 113 filles), âgés de 4 à 15 ans, ont été diagnostiqués positifs à *S. haematobium.* Ces 245 enfants infestés ont tous reçu deux doses uniques de 40 mg/kg de PZQ par voie orale à quatre semaines d'intervalle et ont été suivis pendant 12 semaines. Le TRO à 4, 8 et 12 semaines post-traitement était de 57,1 %, 77,6 % et 100 %, respectivement. Il était significativement plus élevé chez les enfants dont l'infestation était légère que chez ceux plus fortement infestés. À 4 semaines post-traitement, la recherche d'œufs était négative chez 121 enfants, dont 1 (6,3) fortement infesté et 120 (52,4 %) plus faiblement. Au cours de la deuxième cure de traitement, le taux de guérison à 8 et 12 semaines était respectivement de 85,3 % et 100 %.

Conclusion : Cette étude a démontré l'efficacité d'une prise orale de PZQ en deux doses pour le traitement de la schistosomiase urinaire parmi des enfants d'âge scolaire au Nigeria.

Translated from English version into French by Suzanne Assenat, through

TRANSLATORS
WITHOUT BORDERS

Эффективность применения празиквантела при лечении инфекционных заболеваний, вызванных *Schistosoma haematobium* среди детей школьного возраста в сельских общинах г. Абеокута, Нигерия

Оюронгбе Олусола, Сина-Агбаэ Олавунви Рисяат, Бусари Абасс, Окорие Патрициа Н, Оюронгбе Таиво Адетола, Акинделе Акеем Абиодун

АННОТАЦИЯ

Предыстория: Уже в течение последних двадцати лет химеотерапия с использованием празиквантела (PZQ) лежит в основе лечения мочеполового шистосоматоза. Будучи единственным лекарством, имеющимся в распоряжении более 200 миллионов человек по всему миру, празиквантел требует постоянного контроля эффективности своего применения, особенного с точки зрения широкого применения данного лекарства.

Методы: Предметом исследования стала эффективность перорального приёма двух доз PZQ для лечения *Schistosoma haematobium (мочеполового шистосоматоза)* среди детей школьного возраста в Нигерии. Были собраны и затем, с применением фильтрационной методики, исследованы образцы мочи у 350 детей школьного возраста. Были взяты анализы крови для изучения на предмет гаматокритного числа (PCV), также в расчёт принимались рост и масса каждого ребёнка. Между тем ученики, с положительными результатами анализов на зародыши *S. haematobium* прошли курс лечения, состоящий в приёме двойной дозировки PZQ из расчёта 40 мг/кг с интервалом в четыре недели. Оценка эффективности лекарственного средства производилась на основе степени снижения объёма обнаруженных зародышей возбудителя (ERR).

Результаты: Среди 350 детей школьного возраста, *S. haematobium (мочеполового шистосоматоз)* был выявлен у 245 (70.0%) – из которых 132 были лицами мужского пола, а 113 - лицами женского пола, при возрастном диапазоне обследуемых от 4 до 15 лет. Все 245 заражённых детей перорально принимали разовую дозу PZQ из расчёта 40 мг/кг два раза с интервалом в четыре недели при продолжительности курса в 12 недель. По истечении четырёх, восьми и двенадцати недель после лечебного периода, выявленная степень ERR равнялась 57,1%, 77,6% и 100% соответственно. Данный показатель у детей с лёгкой степенью инфицирования был значительно выше по сравнению с детьми, подвергшимися тяжёлой степени заражения. По истечении

четырёх недель после лечебного периода отрицательные результаты на наличие зародыша возбудителя были получены в отношении ста двадцати одного ребёнка, среди которых 1 (6.3%) исследуемый страдал тяжёлой степенью заражения, тогда как 120 (52.4%) - лёгкой степенью инфицирования. По окончании второй стадии лечения, показатель эффективности лечения был равен 85,3% и 100% после восьми и двенадцати недель соответственно.

Заключение: Данное исследование продемонстрировало эффективность перорального приёма двух доз празиквантела (PZQ) для лечения мочеполового шистосоматоза среди детей школьного возраста в Нигерии.

Translated from English version into Russian by Mikhail Abramkin, through

TRANSLATORS
WITHOUT BORDERS

Eficacia de Efficacy of praziquantel en el tratamiento de la infección *Schistosoma haematobium* entre niños en edad escolar de las comunidades rurales de Abeokuta, Nigeria.

Ojurongbe Olusola, Sina-Agbaje Olawunwi Risqat, Busari Abass, Okorie Patricia N, Ojurongbe Taiwo Adetola, Akindele Akeem Abiodun

RESUMEN

Antecedentes: La quimioterapia con praziquantel (PZQ) ha sido la piedra angular en el control del esquistosoma durante más de dos décadas. Al ser el único medicamento disponible para el tratamiento de aproximadamente 200 millones de personas en todo el mundo, es recomendable ejercer un seguimiento continuo a la eficacia del PZQ bajo la presión de su uso difundido.

Métodos: La eficacia de consumir dos dosis de PZQ de forma oral para el tratamiento del *Schistosoma haematobium* fue evaluada entre niños en edad escolar en Nigeria. Se recolectaron 350 muestras de orina de niños en edad escolar, las cuales fueron examinadas mediante la aplicación de la técnica de filtración. También se recolectó sangre para la estimación del volumen corpuscular medio (VCM), y se chequeó el peso y la altura de cada niño. Los alumnos que resultaron positivos al *S. haematobium* fueron tratados con dos dosis orales de PZQ de 40 mg/kg con un intervalo de cuatro semanas entre sí. La eficacia del medicamento se determine en base a la tasa de reducción de huevos (TRH).

Resultados: Entre 350 niños en edad escolar, 245 (70.0%) – de los cuales 132 fueron varones y 113 fueron hembras, con edades que oscilaban entre los cuatro y los 15 años – fueron diagnosticados con *S. haematobium.* Todos los 245 niños infectados recibieron una dosis simple de forma oral de 40 mg/kg de PZQ dos veces con un intervalos de cuatro semanas y con seguimiento durante 12 semanas. En la cuarta, octava y doceava semanas siguientes al tratamiento, la TRH fue de 57.1%, 77.6% y 100%, respectivamente. La TRH fue significativamente mayor entre niños con una infección leve en comparación con aquellos con una infección fuerte. Ciento veintiún niños resultaron negativos a las cuatros semanas siguientes al tratamiento, entre los cuales 1 (6.3) y 120 (52.4%) tenían infecciones leves y fuertes, respectivamente. Posterior a la segunda ronda de tratamiento, la tasa de curación a las ocho semanas y 12 semanas fue de 85.3% y 100%, respectivamente.

Conclusión: Este estudio demostró la eficacia de la administración de dos dosis orales de PZQ para el tratamiento del esquistosoma urinario entre los niños en edad escolar de Nigeria.

Translated from English version into Spanish by SoyBienestar, through

TRANSLATORS
WITHOUT BORDERS
